# Tiered Evaluation of Carbosilane Dendrimer–siRNA Nanoplatform from Single-Cell Biocompatibility to Blood-Brain Barrier Model Dynamics and Murine Alzheimer Model Behavior Assessment

**DOI:** 10.1021/acsami.6c06099

**Published:** 2026-07-06

**Authors:** Serafin Zawadzki, Elżbieta Okła, Sylwia Michlewska, Radosław Bednarek, Paula Ortega López, F. Javier de la Mata, Juris Jansons, Dace Skrastina, Madara Kreišmane, Vladimirs Piļipenko, Baiba Jansone, Maksim Ionov, Maria Bryszewska, Katarzyna Miłowska

**Affiliations:** 1 Department of General Biophysics, Faculty of Biology and Environmental Protection, University of Lodz, 141/143 Pomorska St., Lodz 90-236, Poland; 2 Bio-Med-Chem Doctoral School of the University of Lodz and Lodz Institutes of the Polish Academy of Sciences, University of Lodz, Matejki 21/23, Lodz 90-237, Poland; 3 Laboratory of Microscopic Imaging and Specialized Biological Techniques, Faculty of Biology and Environmental Protection, 49602 University of Lodz, Banacha 12/16, Lodz 90-237, Poland; 4 Department of Cytobiology and Proteomics, 37808 Medical University of Lodz, Lodz 92-215, Poland; 5 Department of Organic and Inorganic Chemistry, IQAR, University of Alcalá, Madrid 28805, Spain; 6 Networking Research Center on Bioengineering, Biomaterials and Nanomedicine (CIBER-BBN), Madrid 28029, Spain; 7 Ramón y Cajal Health Research Institute (IRYCIS), Madrid 28034, Spain; 8 Latvian Biomedical Research and Study Centre, Ratsupites str 1 k-1, Riga 1067, Latvia; 9 Department of Neuromedicine and Neuroscience, Faculty of Medicine and Life Sciences, University of Latvia, Jelgavas street 3, Riga 1067, Latvia; 10 Faculty of Medicine, Collegium Medicum, Mazovian Academy in Plock, 2 Dabrowskiego Sq, Plock 09-402, Poland

**Keywords:** carbosilane dendrimer, nanoparticles, siRNA, blood−brain barrier model, behavioral assessment, Alzheimer’s disease

## Abstract

Blood–brain barrier (BBB) transport remains a primary constraint on achieving predictable central nervous system exposure for Alzheimer’s disease (AD) therapeutics, motivating the evaluation of delivery platforms with barrier-resolved and functionally relevant end points. We assessed a carbosilane dendrimer (G3Si PEG6000) and its siRNA dendriplex using a tiered, upstream strategy spanning cell internalization, DNA damage screening, BBB model integrity and permeability, and *in vivo* AD-relevant murine model learning. At the cellular level, the dendrimer enhanced intracellular siRNA-associated signal with predominantly cytoplasmic localization, and siRNA complexation attenuated genotoxicity relative to the noncomplexed carrier. In a BBB triculture model, barrier function was preserved without sustained transendothelial electrical resistance (TEER) loss, and complementary tracer flux readouts showed time- and formulation-dependent, nonmonotonic changes, including TEER–permeability decoupling consistent with nonuniform perturbation and time-dependent changes in barrier-associated paracellular responses. In APOE4 knock-in mice, dendriplex treatment increased platform-zone crossings in the Morris Water Maze probe trial, whereas target-quadrant time showed only a modest, nonsignificant trend. Collectively, these integrated results indicate that siRNA complexation improves the BBB-relevant safety-performance balance of G3Si PEG6000 and supports further studies that directly link brain exposure and target engagement to cognitive outcomes.

## Graphical abstract

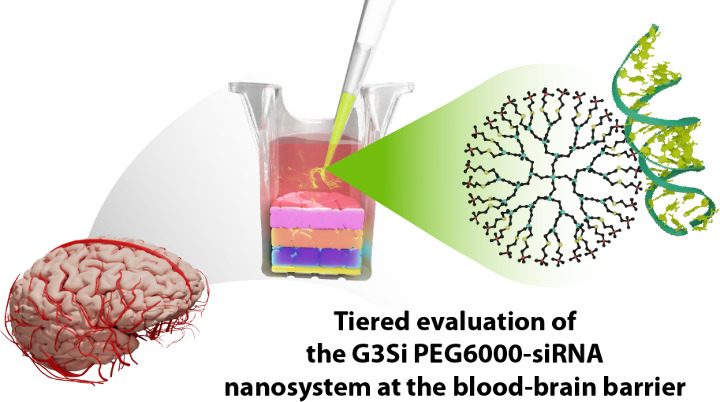

## Introduction

Contemporary models describe Alzheimer’s disease (AD) as a multifactorial disorder in which genetic susceptibility (e.g., APOE and APP variants) and environmental or aging-related stressors converge on partially overlapping biological cascades that ultimately drive synaptic failure, progressive neurodegeneration, and dementia. In the amyloid-centered pathway, amyloid precursor protein (APP) is cleaved sequentially by beta-site APP cleaving enzyme 1 (BACE1) and gamma-secretase, generating amyloid-beta (Aβ) peptides that can oligomerize and deposit as extracellular plaques. In parallel, Aβ and APP-derived fragments are proposed to amplify kinase and phosphatase imbalance and inflammatory signaling, thereby promoting tau hyperphosphorylation, secretion, cellular uptake, and propagation along synaptically connected neuronal pathways. These processes convert tau from a microtubule-stabilizing protein into an aggregation-prone species that forms neurofibrillary tangles (NFTs) and disrupts axonal and synaptic communication. Together, amyloid- and tau-linked pathways, coupled with chronic neuroinflammation and vascular dysfunction, contribute to synaptic failure, neuronal loss, brain atrophy, resulting in clinical dementia that primarily manifests as memory loss, aphasia, agnosia, visuospatial impairment, deficits in critical thinking and calculation, along with personality and behavioral changes.^1,2^ An increasingly supported hypothesis posits that early cerebrovascular impairment, particularly the blood-brain barrier (BBB) dysfunction, acts as an upstream contributor to the disease continuum rather than merely a downstream consequence. Multiple independent lines of evidence identify BBB breakdown as an early vascular abnormality in AD, documented by neuroimaging and corroborated by both animal models and post-mortem human tissue analyses.^3–8^ In this context, the two-hit vascular hypothesis proposes that an initial insult driven by vascular risk factors and aging results in BBB disruption, capillary hypoperfusion, and impaired microvascular function, thereby permitting the entry of blood-derived neurotoxic/pro-inflammatory factors into the brain. This altered milieu is then proposed to facilitate a second insult marked by Aβ accumulation, tau pathology, and progressive neurodegeneration.^9,10^

Despite major advances in mechanistic understanding, approved pharmacotherapies remain largely symptomatic. Acetylcholinesterase inhibitors (donepezil, rivastigmine, galantamine) reversibly increase synaptic acetylcholine and transiently enhance cholinergic signaling. These agents can yield modest improvements or short-term stabilization in cognition and daily function for some patients, but they do not modify underlying disease biology.^11^ Memantine, an *N*-methyl-d-aspartate (NMDA) receptor antagonist approved for moderate-to-severe AD, acts as an uncompetitive, voltage-dependent blocker that preferentially attenuates sustained pathological NMDA receptor activation and calcium-mediated excitotoxicity while largely preserving physiological glutamatergic transmission.^12^ Consequently, current standard-of-care medicines can provide limited symptomatic benefit but do not halt disease progression. In contrast, the clinical trial landscape spans multiple mechanistic-centered strategies, including therapies directed at neuroinflammation, synaptic resilience and neuroprotection, oxidative injury, and amyloid- and tau-linked processes. As a snapshot of the ClinicalTrials.gov registry on February 2026 identified 164 active, interventional AD studies across Phases 1 to 4, with 13 listed as Phase 1, 38 as Phase 2, 22 as Phase 3, and 6 as Phase 4. Within the field, anti-Aβ monoclonal antibodies such as aducanumab (Aduhelm), lecanemab (Leqembi), and donanemab (Kisunla) have received accelerated or conditional approvals, representing the first wave of FDA-approved anti-Aβ immunotherapeutics.^13–15^ The antiamyloid beta monoclonal antibodies reduce plaque burden and have yielded statistically significant modest clinical slowing in early AD, yet analyses of their pharmacokinetics emphasize that the blood-brain barrier severely limits effective CNS exposure. The aducanumab was reported to cross the BBB because brain concentrations appeared to track plasma levels in mice, yet the reported brain-to-plasma ratio of approximately 1 μL/g is more than 10-fold lower than the estimated cerebral plasma volume of about 10 to 15 μL/g.^16,17^ This quantitative mismatch implies that much of the measured signal can be explained by antibody confined to the residual blood volume rather than parenchymal penetration. Consequently, without correcting for residual blood volume, brain uptake estimates risk overestimating effective delivery across the BBB and may foster the misconception that no dedicated BBB transport technology is required for CNS biologics. Rational therapeutic design in AD increasingly shifted toward strategies that explicitly incorporate the BBB not as a passive backdrop but as a central design parameter.

Accounting for this complexity, BBB critically shapes not only whether therapeutics enter the CNS, but also how they distribute within brain tissue. Accordingly, explicit consideration of BBB architecture and CNS compartmentalization should be central to therapeutic design. Contrary to the intuitive assumption that disease-driven BBB disruption would passively enhance access of therapeutics to degenerating brain regions, vascular injury and BBB pathology introduce profound hindrance to effective drug distribution. Reported features of neurodegenerative vascular injury include endothelial degeneration, altered tight and adherens junction protein expression, increased nonspecific transcytosis, transporter dysregulation, pericyte loss, perivascular accumulation of blood-derived proteins, and sustained neuroinflammation, all of which can perturb solute handling and tissue microenvironment properties.^18–20^ Under these conditions, water and solutes accumulate in enlarged perivascular spaces, impairing normal interstitial fluid formation, convection and diffusion through the extracellular space, so that exogenous therapeutics tend to be sequestered with blood-borne debris rather than reaching neuronal targets at effective concentrations.^21,22^ In addition, dysfunction or downregulation of carrier-mediated and receptor-mediated transport pathways can further limit delivery for substrates that rely on these routes.^18^ Consequently, while transient and spatially controlled BBB opening, for example by focused ultrasound in the presence of intravenously administered microbubbles, can enhance regional drug or gene delivery in selected settings such as brain tumors, chronic or diffuse BBB disruption appears to promote neurodegenerative cascades and compromise therapeutic distribution rather than a reliable facilitator of delivery.^23,24^

Considering the increasing recognition that BBB dysfunction can act as an upstream contributor to neurodegeneration, we prioritized targets that mechanistically link Alzheimer’s disease risk to neurovascular unit pathology. Multiple AD-associated risk genes have functions relevant to vascular biology, endocytosis, and barrier maintenance (e.g., PICALM, BIN1, CD2AP, RIN3, FMNL2), supporting BBB-oriented target selection.^25,26^ In particular, APOE ε4 is the strongest common genetic risk factor for late-onset AD and has been associated with structural and functional vascular abnormalities, including altered basement membrane morphology and leakage of plasma-derived proteins in affected brain regions, with evidence that these changes can contribute to blood flow dysregulation and cognitive decline independent of canonical AD lesions.^4,27,28^ Mechanistic studies report that APOE ε4 can promote BBB breakdown, reduce cerebral blood flow, and contribute to neuronal injury independently of Aβ, in part through pericyte-linked cyclophilin A and matrix metalloproteinase 9 signaling with downstream effects on junctional proteins such as ZO-1 and occludin, supported by complementary findings in post-mortem tissue and cerebrospinal fluid biomarkers in ε4 carriers.^8,28,29^ In parallel, the APOE ε4 isoform can impair amyloid homeostasis through receptor-mediated mechanisms that slow Aβ clearance.^30^ Together, these lines of evidence motivate our allele-selective RNA interference (RNAi) approach in which siRNA is used to preferentially silence apolipoprotein E4 (APOE4), with the goal of mitigating AD-relevant pathology at the neurovascular interface and within the brain parenchyma. To address this question in a translationally relevant framework, we evaluated G3Si PEG6000 and its siRNA dendriplex across coordinated cellular, BBB-model, and murine functional readouts. We hypothesized that complexation of siRNA with G3Si PEG6000 would yield a more favorable biological profile in cellular and BBB-model studies and support exploratory *in vivo* functional evaluation of the dendriplex in an AD-relevant murine model.

In this context, nanoparticle platforms offer modular carriers whose size, architecture, and surface chemistry can be rationally engineered guided by BBB permeability and its underlying architecture and physiology, enabling therapeutic strategies to influence both the BBB and brain parenchyma. Surface ligands can be used to engage receptor- or carrier-mediated transcytosis, while cationic or amphiphilic coatings may support adsorptive-mediated transcytosis.^31^ A broad range of nanomaterials has been explored for central nervous system delivery, including dendrimers, polymeric and metallic nanoparticles, nanoemulsions, nanocrystals, and lipid-based systems, with the shared goal of achieving therapeutically relevant brain exposure. Among these, dendrimer nanoparticles have gained attention for their high versatility and structurally well-defined nanocarriers, consisting of a symmetrical core with radially branching repeat monomer units that define the generation number.^32,33^ With the nanoformulation design constraints imposed by BBB architecture in mind, we synthesized a third-generation carbosilane dendrimer with a silicon core and PEG6000 graft (Dendrimer G3Si PEG6000). This nanosystem is designed to provide a size distribution compatible with transcytosis-based transport mechanisms and a high density of terminal groups suitable for conjugation or complexation of nucleic acid cargo while limiting excessive surface charge that could compromise biocompatibility. PEGylation is intended to improve colloidal stability in biological media, reduce opsonisation and nonspecific protein binding, and prolong systemic circulation, thereby increasing the probability of brain exposure while limiting peripheral off-target interactions.^34^

These design expectations require validation through a decision-guided, multimethod validation framework that integrates and translates evidence from multiple sources into transparent safety–performance conclusions. We first established the nanocarrier’s synthetic identity in prior work by comprehensively characterizing the carbosilane dendrimer nanosystem biophysical parameters and evaluating interactions with model proteins to anticipate formation of a biologically relevant biomolecular corona, together with hemocompatibility testing using recognized practices for blood-contacting materials.^35–37^ Our study was conducted under a weight-of-evidence intelligent testing strategy and an integrated approach to testing and assessment (ITS/IATA) framework, in line with OECD guidance and ISO/TR 16197 recommendations for toxicological screening of manufactured nanomaterials. Building on that foundation, the present article integrates a coordinated end point set spanning genotoxicity assessment, cellular uptake, BBB model integrity assessed by transendothelial electrical resistance (TEER), BBB paracellular permeability, spatial learning, and memory in an Alzheimer’s disease murine model. Collectively, this structured framework is intended to support a transparent, fit-for-purpose appraisal of the nanocarrier’s biological interactions, BBB-relevant performance, and *in vivo* functional readouts within a single, reproducible assessment framework (Figure 1).

**Figure 1:**
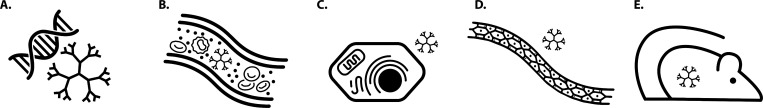
Tiered evaluation strategy of the G3Si PEG6000 dendrimer–siRNA nanoplatform. (A) Prior biophysical evaluation of the dendrimer and dendriplex, including characterization of complexation behavior, size/charge-related properties, morphology, and siRNA-binding effects.^35^ (B) Prior blood-interaction assessment, including protein interaction and hemocompatibility profiling.^36,37^ (C) Single-cell analysis, (D) BBB model interactions, and (E) murine Alzheimer model evaluation represents the tiers addressed in the current manuscript. Within these tiers, the present study includes set of assessments cellular uptake and DNA-damage screening at the single-cell level, barrier-integrity and permeability-related measurements in the BBB model, and behavioral assessment in the murine model. The overall framework follows a tiered, weight-of-evidence strategy progressing from prior nanocarrier characterization through cellular and BBB-relevant testing to animal-level evaluation.

## Materials and Methods

### Dendrimer

Dendrimer with a silicon atom core, surface tertiary ammonium groups, and single polyethylene glycol (PEG) (6000) – G3Si PEG6000 with chemical formula C_601_H_1324_I_30_N_31_O_135_S_32_Si_29_ and molar weight of 16794.81 g/mol is presented in Figure 2. The studied dendrimer was diluted in a phosphate buffer solution of 7.4 pH. The synthesis and biophysical characterization of the dendrimer, along with its dendriplex with siRNA, was described in previous work.^35^

**Figure 2:**
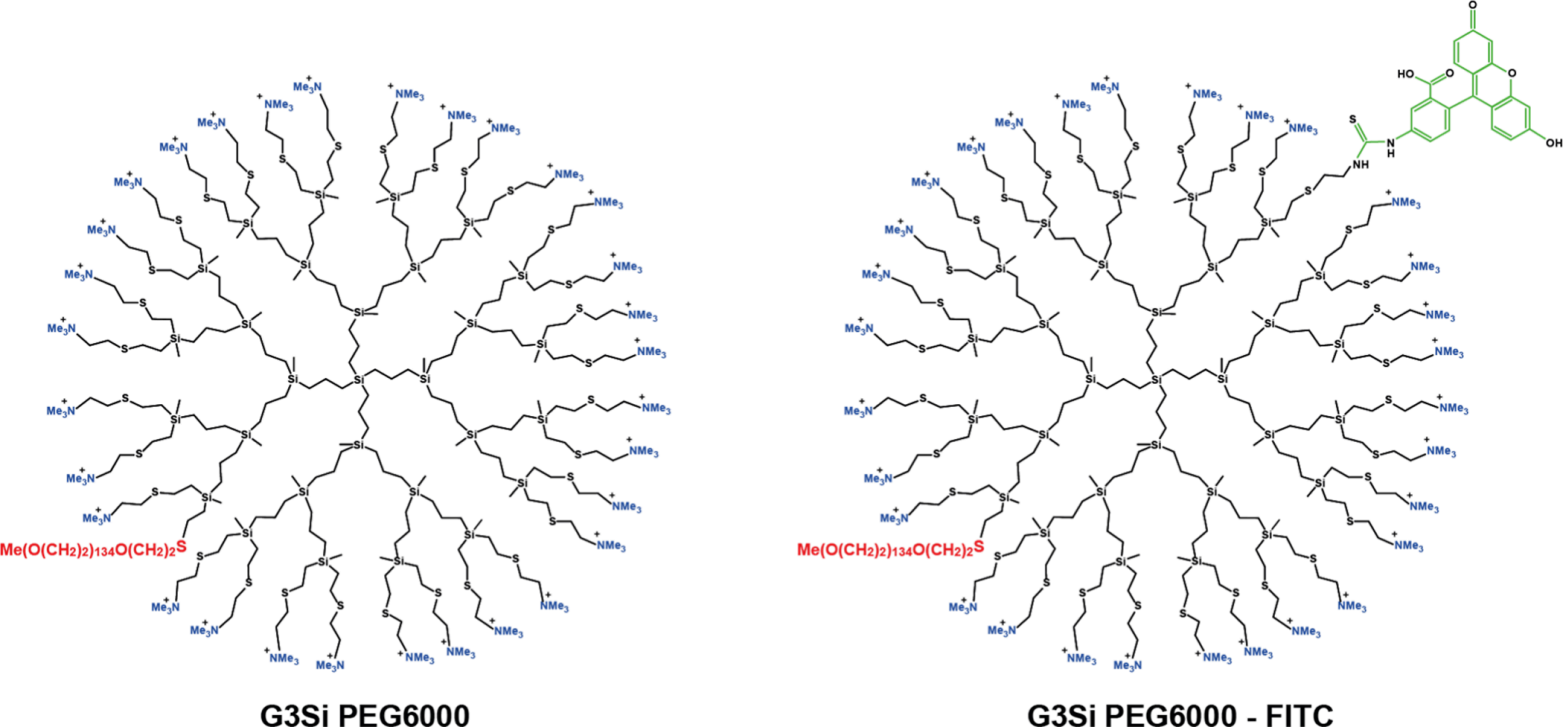
Structure of the studied dendrimer G3Si PEG6000 and its FITC-labeled counterpart.

### siRNA

The following siRNA was used in the present study (Dharmacon Inc., Lafayette, CO, USA):

Sense: 5′-GAUUACCUGCGCUGGGUGCUU
Antisense: 5′-P GCACCCAGCGCAGGUAAUCUU

The following siRNA labeled with Cy5 fluorescence dye was used in the present study (Dharmacon Inc., Lafayette, CO, USA):

Sense: 5′- Cy5 GAUUACCUGCGCUGGGUGCUU
Antisense: 5′-P GCACCCAGCGCAGGUAAUCUU

### Dendriplex Formation

Our prior research provides insights into the dendrimer/siRNA complexation process. We characterized dendrimer G3Si PEG6000 and determined its optimal dendriplex saturation at a molar ratio of dendrimer to siRNA of 2.5:1.^35^ In the present study, dendriplexes were prepared in phosphate-buffered saline (PBS; Sigma-Aldrich, Saint Louis, MO, USA) at this fixed ratio. siRNA was first diluted to the desired concentration in PBS, followed by addition of G3Si PEG6000 under gentle pipet mixing, and the mixtures were incubated for 20 min at room temperature before use. The concentration range applied in the experiments was 0.1–10 μM for G3Si PEG6000 and 0.04–4 μM for siRNA, with both components scaled proportionally so that the 2.5:1 dendrimer:siRNA molar stoichiometry was maintained. Dendriplexes containing fluorescently labeled components were prepared in the same manner, with handling performed in the dark. The current work was performed using a formulation optimized previously, whereas assay-medium-specific physicochemical recharacterization was not conducted in the present study and should be considered when interpreting the biological findings.^35^

### Cell Lines

Human brain microvascular endothelial cells (HBEC-5i; ATCC, Manassas, VA, USA) were cultured on tissue culture flasks precoated with 1% gelatin in Dulbecco’s Modified Eagle Medium/Nutrient Mixture F-12 (DMEM/F-12; Biowest, Nuaillé, France) supplemented with 10% heat-inactivated fetal bovine serum (FBS; Biowest, Nuaillé, France), 1% penicillin–streptomycin (P/S; Sigma-Aldrich, Saint Louis, MO, USA), endothelial cell growth supplement (ECGS; Sigma-Aldrich, Darmstadt, Germany), and 1 μg/mL hydrocortisone (MP Biomedicals, Santa Ana, CA, USA). Cells were maintained at 37 °C in a humidified incubator with 5% CO_2_.

Human brain vascular pericytes (HBVP; ScienCell Research Laboratories, Carlsbad, CA, USA) were cultured on flasks coated with 2 μg/cm^2^ poly-l-lysine (PLL) in pericyte medium supplemented with FBS, pericyte growth supplement (PGS), and P/S (all from ScienCell Research Laboratories, Carlsbad, CA, USA). Cells were maintained at 37 °C in a humidified incubator with 5% CO_2_.

Human astrocytes (HA; ScienCell Research Laboratories) were grown on flasks coated with 2 μg/cm^2^ PLL in astrocyte medium supplemented with FBS, astrocyte growth supplement (AGS), and P/S (all from ScienCell Research Laboratories, Carlsbad, CA, USA). Cells were maintained at 37 °C in a humidified incubator with 5% CO_2_.

### Genotoxicity Assessment

DNA strand-break–associated damage in HBEC-5i cells was screened using the Fast Halo Assay (FHA) as described by Sestili et al. The assay quantifies radial diffusion of alkali-solubilized DNA fragments from nucleoids at the single-cell level following alkaline extraction and fluorescent DNA staining.^38^ Cells were seeded into 24-well plates at 5 × 10^4^ cells/well and cultured for 24 h (37 °C, 5% CO2). To evaluate the effects of G3Si-PEG6000 and its dendriplex, cells were exposed to each formulation for 24 h. Untreated cells as the negative control, and 10 μM H_2_O_2_ was included as a positive control to confirm assay responsiveness and was processed in parallel through the same FHA workflow. Cells were collected by trypsinization, resuspended in cold PBS, washed twice, and embedded in a 1% low-melting-point agarose solution (Sigma-Aldrich, Darmstadt, Germany). A small amount of the cell suspension was applied to a glass slide precoated with normal-melting-point agarose (Sigma-Aldrich, Darmstadt, Germany) and covered with a coverslip. After the agarose had solidified on ice, the coverslip was removed, and the slides were incubated for 15 min in 0.3 M NaOH (pH ≥ 13.0) at room temperature. Ethidium bromide was added during the final 5 min to a final concentration of 25 μg/mL, followed by destaining in distilled water for up to 15 min. Slides were immediately examined under a Zeiss Axio Scope A1 fluorescence microscope (Carl Zeiss, Germany) with exposure settings fixed across conditions. Images were analyzed using ImageJ software and HaloJ plugin.^39^ The results are expressed as the nuclear diffusion factor (NDF), which is the ratio of the total area of the halo plus the nucleus to the area of the nucleus. The experiment was performed at least 4 repetitions.

### Cellular Uptake by Confocal Microscopy

HBEC-5i cells were seeded at 1 × 10^4^ cells per well in glass-bottom imaging plates (Ibidi GmbH, Gräfelfing, Germany) and cultured under standard conditions (37 °C, 5% CO_2_) for 24 h. Cells were then incubated for 3 or 24 h with the G3Si PEG6000 dendrimer labeled with FITC, the siRNA labeled with Cy5, and their dendriplex prepared from FITC-labeled G3Si PEG6000 and Cy5-labeled siRNA. After incubation, cells were washed with PBS, fixed with 4% formaldehyde (Avantor Performance Materials Poland S.A., Gliwice, Poland), followed by washing with PBS and staining with DAPI (4′,6-diamidino-2-phenylindole) (Thermo Scientific TM, ThermoFischer Scientific, Waltham, MA, USA) at 0.5 μg/mL for 5 min.

Images were acquired on a Leica TCS SP8 confocal microscope (Leica Microsystems, Wetzlar, Germany) equipped with a 63×/1.40 oil objective (HC PL APO CS2, Leica Microsystems, Germany). Fluorescence was acquired using the following channel settings: DAPI (405 nm excitation, 430–470 nm emission), FITC (489 nm excitation, 500–530 nm emission), and Cy5 (650 nm excitation, 660–720 nm emission).

### Blood–Brain Barrier Model Integrity by Transendothelial Electrical Resistance (TEER)

A human triculture BBB model comprising astrocytes, pericytes, and brain endothelial cells was established on permeable membrane inserts (ThinCert, Greiner Bio-One) with 1.0 μm pores and a membrane growth area of 33.6 mm^2^ in 24-well plates. Inserts were precoated with poly-l-lysine (PLL, 10 μg/mL; 50 μL per insert; 3 h), then rinsed twice with sterile water. Astrocytes were seeded first at 2.0 × 10^4^ cells/cm^2^, allowed to attach for 30 min at room temperature, and subsequently maintained at 37 °C and 5% CO_2_ for 48 h. Pericytes were then added at 2.0 × 10^4^ cells/cm^2^ and cultured for an additional 48 h. Finally, HBEC-5i endothelial cells were seeded at 8.0 × 10^4^ cells/cm^2^ to complete the triculture (Figure 3). From the time of endothelial seeding onward, cultures were maintained in complete HBEC-5i medium supplemented with amphotericin B (1 μg/mL), with medium exchanges every 48 h in both apical and basolateral compartments. G3Si PEG6000 dendrimer and its dendriplex were added on the 13th day of the experiment, once a stable plateau had formed. Monitoring continued at 24 h intervals, with medium exchanges every 48 h only in the basolateral compartments. TEER was measured using an EVOM3 epithelial volt-ohmmeter (World Precision Instruments, FL, USA) connected to an EndOhm-6G chamber electrode. The measurement principle is based on a four-electrode configuration in which a known constant current is driven through the sample on one electrode pair and the voltage drop is sensed on the other pair, after which resistance is computed from Ohm’s law. For each experiment, blank resistance (R_BLANK_) was measured using coated inserts containing medium but no cells, capturing background contributions. The total resistance across cell-seeded inserts (R_TOTAL_) comprises the cell-layer component together with these background terms, and the cell-specific resistance (R_TISSUE_) was obtained by subtraction:

$$R_{TISSUE}=R_{TOTAL}-R_{BLANK}$$

**Figure 3:**
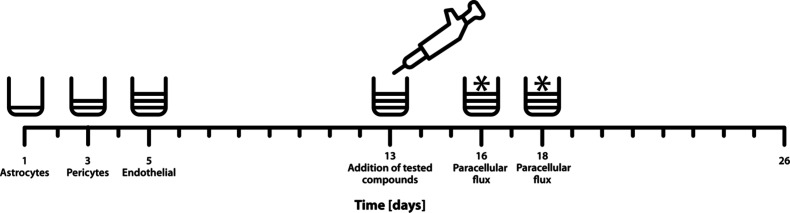
Schematic overview of the experimental schedule for the BBB model. Astrocytes were seeded on day 1, followed by pericytes on day 3 and brain microvascular endothelial cells on day 5. The BBB model was allowed to mature until day 13, when the G3Si PEG6000 dendrimer or its siRNA dendriplex was added. Permeability of 40 kDa FITC-dextran through the BBB model was measured on days 16th and 18th.

TEER was then area-normalized and reported as TEER_REPORTED_ in Ω·cm^2^ using the equation:

$${TEER}_{REPORTED}=R_{TISSUE}-M_{AREA}$$

where M_AREA_ is the membrane growth area in cm^2^. Accordingly, TEER_REPORTED_ represents the area-averaged, blank-corrected electrical resistance across the cell layer on the inset and is inversely related to net ionic conductance across the cell barrier. The experiment was performed in 3 repetitions.

### Blood–Brain Barrier Paracellular Permeability

The BBB triculture model was established as described above. Paracellular macromolecular permeability was assessed by quantifying the translocation of 40 kDa FITC dextran across the barrier using a Transwell flux assay. G3Si PEG6000 dendrimer and its dendriplex were added on day 13 to the apical compartment, after formation of a stable TEER plateau.

Once a stable TEER plateau had formed, G3Si PEG6000 dendrimer and its dendriplex were added on day 13 to the apical compartment (Figure 3). Permeability measurements were performed 3 and 5 days after compound addition based on longitudinal TEER monitoring indicated that these time points corresponded to an early established and a later developed phase of the postexposure barrier response in the matured BBB model. 40 kDa FITC–dextran (Invitrogen, MA, USA) was added to the upper compartment to a final concentration of 400 μg/mL. Receiver-well fluorescence was read directly in the black 24-well plate without sampling at t = 0 and 15, 30, 45, 60, 90, and 120 min using a BioTek microplate reader (BioTek Instruments Inc., VT, USA) with excitation at 485 nm and emission at 530 nm. In parallel, cell-free coated inserts containing medium but no cells were included to define membrane/background tracer passage under identical assay conditions and to confirm that the measured fluorescence signal was barrier-dependent. Permeability was expressed as a percentage of the untreated control at each time point according to

$$\%Permeability=\left(\frac{F_{s}}{F_{c}}\right)\times 100\%$$

Where F_s_ is the fluorescence of cells treated with dendrimer or dendriplex, and F_c_ is the fluorescence of control cells (untreated). The experiment was performed in 3 repetitions.

### Animals

All procedures complied with the bioethical principles adopted by the European Convention for the Protection of Vertebrate Animals Used for Experimental and Other Scientific Purposes (Strasbourg, 1986), Order of the Ministry of Health of the Russian Federation of 23 August 2010 “Establishment of the Rules of Laboratory Practice” No. 708n. The ethical permit Nr. 142/2023 for the described experiments was obtained from the Latvian Food and Veterinary Service Ethics Commission. Female C57BL/6 mice (8–10 weeks; Charles River, Germany) and APOE4 knock-in (APOE4 KI) mice (B6­(SJL)-*Apoe* ^tm1.1(APOE*4)Adiuj^/J; 8–10 weeks; The Jackson Laboratory) were group-housed (5–8 per cage) in environmentally enriched cages at 22 °C on a 12 h light/12 h dark cycle with ad libitum access to food and water. Animals were regularly monitored for food and water intake, weight gain, skin and fur changes, and microscopic alterations at the injection site. All animals were acclimatized for 1 week before the experiments began. For invasive procedures, mice were anesthetized with isoflurane in oxygen (induction 4%, maintenance 2.3%) delivered via facial masks or by ether inhaled from the gauze-soaked nose cones, with peri-procedural monitoring; analgesia was provided as indicated by the procedure-specific welfare assessment. Mice were humanely euthanized by cervical dislocation following deep anesthesia. Procedures were performed by trained personnel.

### Spatial Learning and Memory in Alzheimer’s Disease Murine Model

Spatial learning and reference memory were assessed at the experimental end point using the Morris Water Maze (MWM). Female APOE4 KI mice and C57BL/6 mice were assigned to the following experimental groups (n = 12 per group): 1) APOE4 KI mice treated with G3Si-PEG6000 dendriplex, 2) APOE4 KI mice treated with naked anti-APOE4 siRNA, 3) APOE4 KI mice treated with PBS, and 4) C57BL/6 mice treated with PBS. Beginning at 8 weeks of age, mice received once-weekly injections for 63 days (nine total injections) according to group allocation, after which behavioral testing was performed. The MWM consisted of a circular pool (diameter 150 cm, height 60 cm) filled with water maintained at approximately 25 °C and rendered opaque to conceal the escape platform. A submerged 10 × 10 cm platform was positioned ∼2 cm below the water surface in a fixed target quadrant. Stationary geometric cues were placed along the maze’s cardinal axes and remained constant throughout the experiment. Animal trajectories were recorded using an overhead camera connected to EthoVision XT 11 software (Noldus, The Netherlands). Spatial learning tests were performed over three consecutive days with two daily sessions separated by 4 h. For each trial, the mouse was released from a pseudorandom start position in a quadrant that did not contain the platform. If a mouse failed to locate the platform within 60 s, the trial ended, and the mouse was guided to the platform and allowed to stay on it for 15 s. Escape latency, the time elapsed since the mouse was submerged in the water until it found and climbed onto a hidden platform, was quantified. A probe trial was performed 24 h after the final spatial learning test with the platform removed. Probe trial performance was quantified as time spent in the target quadrant and the number of crossings across the former platform zone.

### Statistical Analyses

Statistical analyses and data visualization were performed using Prism version 10.4.2 (GraphPad Software, Boston, MA, USA). The Shapiro–Wilk test was used to assess the normality of data distributions. For factorial designs, multiple-way ANOVA was applied to assess differences in group means. Where ANOVA indicated statistically significant main effects, appropriate post hoc multiple comparison tests were selected based on the structure of the comparisons: Tukey’s test was used for evaluating all pairwise group differences, Dunnett’s test for comparisons of multiple treatments against a single control group, and Šidák’s test for a limited set of planned comparisons. For non-normally distributed data or ordinal outcomes, pairwise differences were assessed using the nonparametric Mann–Whitney test, with effect sizes reported as rank-biserial correlation (r_rb_). All tests were two-tailed and performed at a significance level α = 0.05. In our graphical representations, significance is denoted by asterisks: one asterisk (*) indicates a p-value <0.05, two asterisks (**) represent a p-value <0.01, and three asterisks (***) denote a p-value <0.001.

## Results and Discussion

RNA interference (RNAi) emerged from transgene-induced cosuppression in plants and post-transcriptional gene silencing in fungi into a unified mechanism, culminating in the discovery that double-stranded RNA elicits potent, sequence-specific silencing in *Caenorhabditis elegans* and later in *Drosophila* and mammalian systems through small interfering RNAs.^40–44^ Recognition of this mechanism by the 2006 Nobel Prize marked a turning point, and subsequent efforts accelerated the translation of RNAi into therapeutics. This is now reflected in the first wave of FDA-approved siRNA therapeutics, which demonstrate that RNAi is clinically feasible. To date, six siRNA therapeutics have been approved by the FDA, namely patisiran (Onpattro), givosiran (Givlaari), lumasiran (Oxlumo), inclisiran (Leqvio), vutrisiran (Amvuttra), and nedosiran (Rivfloza). These agents must overcome multiple extracellular and intracellular barriers and therefore rely on chemical modification and/or specialized delivery systems to achieve efficient delivery. Patisiran is the first up-to-date FDA-approved only nanocarrier formulation to enable siRNA delivery, whereas subsequent approvals primarily use covalent triantennary N-acetylgalactosamine (GalNAc) conjugation to exploit efficient hepatocyte uptake after subcutaneous dosing.^45,46^ A defining feature of this portfolio is that the clinical success has so far been built around a relatively narrow, hepatocyte-optimized set of delivery solutions. At the same time, the broader RNAi therapeutic landscape has expanded substantially, with 139 (14 active, not recruiting) siRNA-related projects listed on ClinicalTrials.gov as of February 2026, reflecting a diverse and growing pipeline beyond currently approved drugs. Against this backdrop, the present article integrates evidence from single-cell-based assays, through multicellular *in vitro* models, to *in vivo* animal studies, progressively evaluating the therapeutic potential of the G3Si PEG6000 nanosystem for safe and effective delivery of siRNA in the context of CNS disease.

### Cellular Uptake by Confocal Microscopy

Efficient intracellular delivery is a prerequisite for RNAi-mediated gene silencing, making the cellular uptake of the G3Si PEG6000 dendrimer-siRNA nanosystem a critical determinant of its functional relevance. We therefore used confocal microscopy in human brain endothelial cells, labeling the dendrimer with FITC and the siRNA with Cy5, and additionally counterstaining nuclei with DAPI to distinguish cytoplasmic from nuclear accumulation of the internalized carrier and cargo. This approach allows independent quantification of carrier and cargo signals and provides a direct assessment of how complexation influences the effective cellular delivery of siRNA.

In the uptake experiments, we used 2.5 μM FITC–G3Si PEG6000, 1 μM Cy5–siRNA, and the corresponding dendriplex because this carrier concentration preserves the previously established 2.5:1 dendrimer:siRNA molar ratio that enables robust fluorescence detection while minimizing confounding effects from overt cytotoxicity. As expected for carrier-free delivery, Cy5–siRNA alone produced near-background red fluorescence at 3 h (0.19 ± 0.17) and low, highly variable redout by 24 h (0.78 ± 1.69) (Figure 4). In contrast, FITC–G3Si PEG6000 alone showed strong, time-dependent green fluorescence (4.46 ± 1.41 at 3 h; 8.81 ± 2.11 at 24 h), indicative of efficient carrier uptake. Complexation of Cy5–siRNA with FITC–G3Si PEG6000 significantly increased red signal relative to free siRNA already at 3 h (dendriplex: red 4.71 ± 2.40; green 4.75 ± 3.01), with a further ∼1.6-fold rise in siRNA-associated red fluorescence at 24 h (7.43 ± 3.34) and a more moderate ∼1.3-fold increase in dendrimer-associated green fluorescence (6.10 ± 2.30). Together, these trends support robust time-dependent cellular uptake of the dendriplex and demonstrate that association with G3Si PEG6000 substantially enhances the effective cellular delivery of Cy5–siRNA compared with the free oligonucleotide. Confocal uptake imaging further showed that, although the bulk of the noncomplexed dendrimer signal remained extranuclear, a small fraction of puncta was detectable within the nuclear compartment, whereas in the dendriplex condition, both Cy5–siRNA and dendrimer fluorescence were restricted to the cytoplasm, consistent with targeting the subcellular domain in which RNAi is executed (Figure 5).

**Figure 4:**
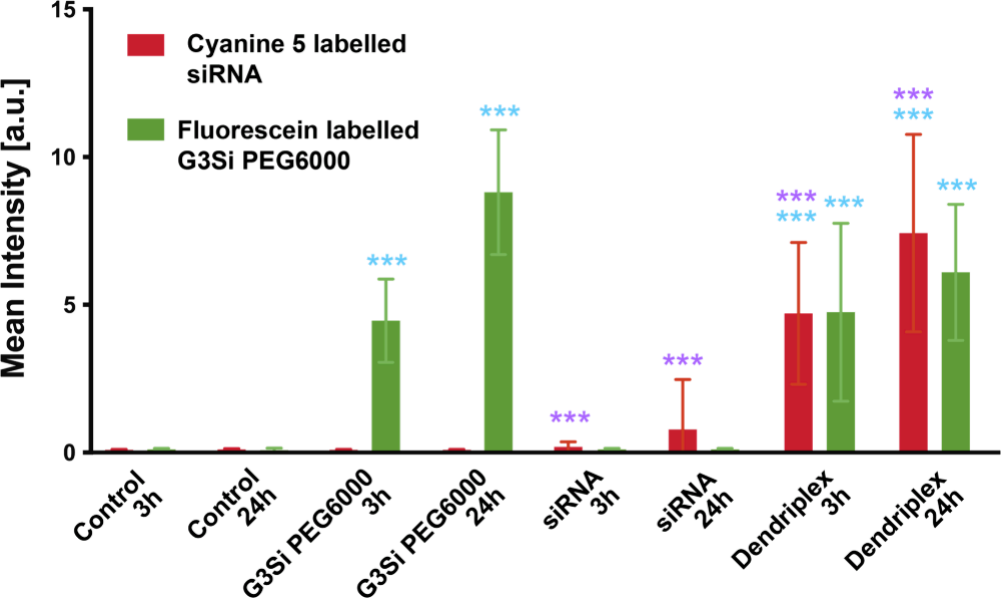
Cellular internalization of 2.5 μM FITC–G3Si PEG6000 dendrimer and 1 μM Cy5–siRNA at 3 and 24 h incubation times. Uptake was quantified as per-cell mean fluorescence intensity (MFI) in the FITC and Cy5 channels. A blue asterisk marks significant differences relative to the control, and a violet asterisk indicates significant differences between the dendrimer and its dendriplex at the corresponding concentration (***p < 0.001). Data are presented as the mean and standard deviation for 5 repetitions.

**Figure 5:**
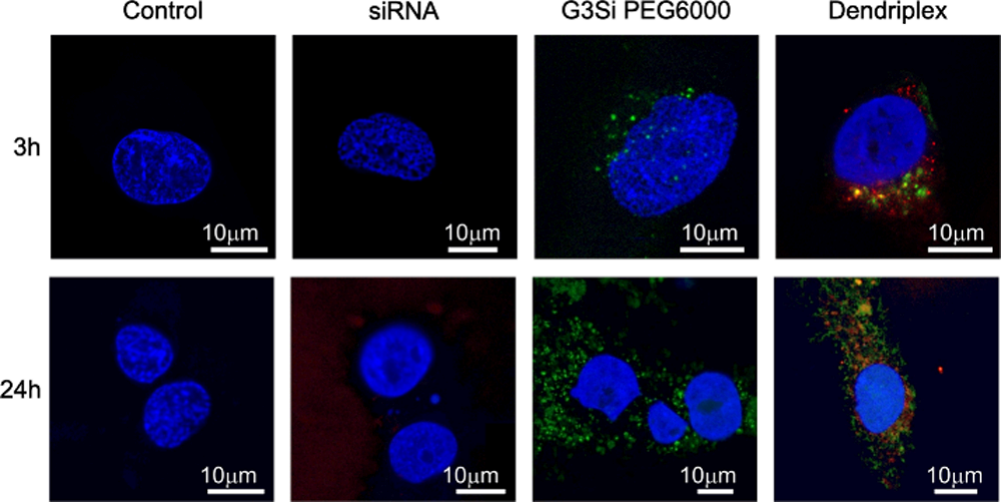
Cellular uptake and subcellular distribution of FITC-G3Si PEG6000 (green) and Cy5–siRNA (red) and their dendriplex in HBEC-5i cells. Representative confocal micrographs of HBEC-5i cells incubated with FITC–G3Si PEG6000 (2.5 μM), Cy5–siRNA (1 μM), or the corresponding dendriplex for 3 or 24 h. Nuclei were counterstained with DAPI (blue). Images were acquired on a confocal microscope using a 63×/1.40 and identical acquisition settings across conditions. Scale bar, 10 μm.

### Genotoxicity Assessment

DNA damage was screened using the Fast Halo Assay, a rapid single-cell nucleoid diffusion method in which DNA radial dispersion and relaxation arising from strand discontinuities, where alkali-labile sites diffuse radially under alkaline conditions and are quantified by image analysis, here reported as the Nuclear Diffusion Factor (NDF). Relative to the negative control (1.16 ± 0.41), cell exposure to the noncomplexed dendrimer produced a pronounced, dose-dependent increase in NDF, reaching 3.2 ± 0.21 at 0.5 μM, 6.91 ± 1.74 at 2.5 μM, and 16.85 ± 0.20 at 10 μM (Figure 6). FHA can capture DNA fragmentation arising from apoptosis or other forms of cell death in addition to genotoxic lesions. This consideration is particularly pertinent at concentrations 2.5 μM and above, which approximates the previously reported IC20 (2.34 μM) for this nanocarrier in the endothelial cell line.^35^ At near-subcytotoxic exposures, even a small fraction of dying cells can disproportionately inflate mean strand-break readouts. Accordingly, we interpret the DNA-damage data in conjunction with cytotoxicity metrics using commonly applied ∼25% cytotoxicity as a pragmatic upper-bound threshold to reduce the risk of false-positive DNA-damage classification.^47^ Notably, a clear FHA signal emerges at 0.5 μM, well below the previously reported IC20 (2.34 μM) for the G3Si PEG6000 nanocarrier, indicating that the response cannot be attributed solely to widespread overt cytotoxicity.

**Figure 6:**
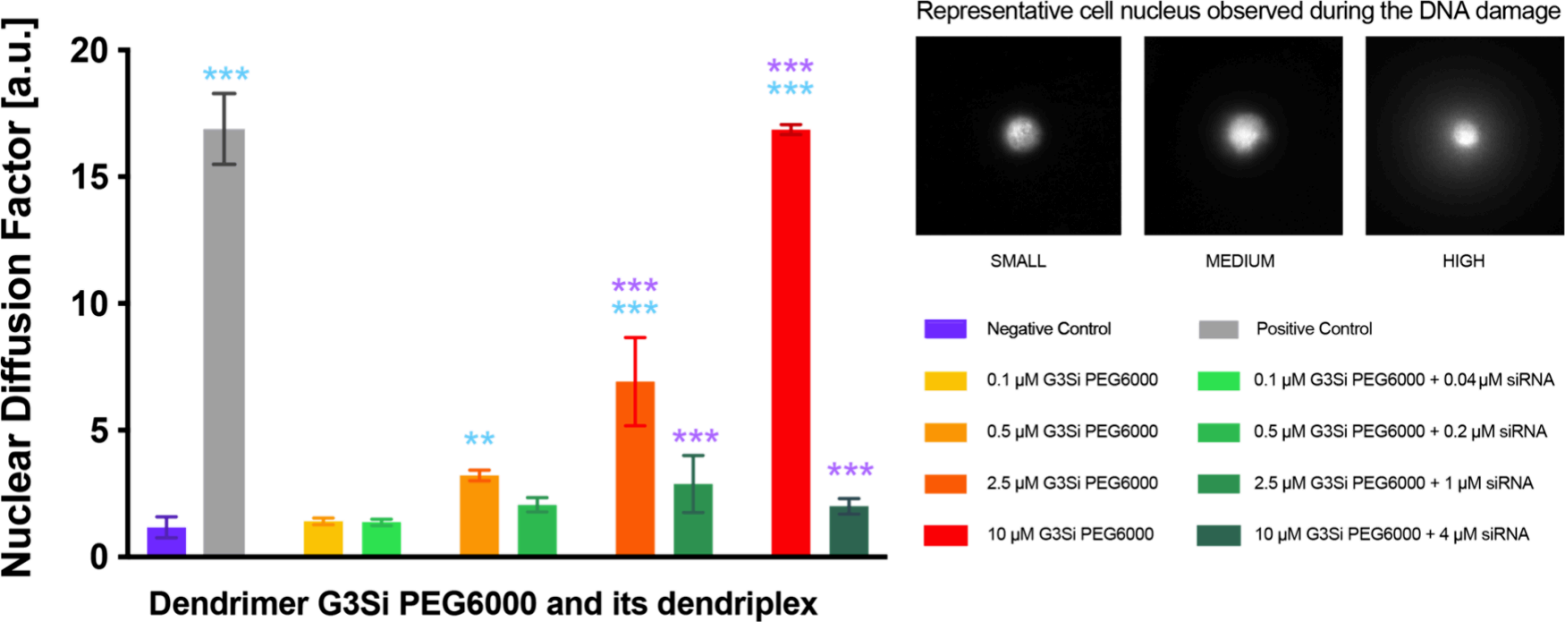
DNA strand-break–associated damage screening by Fast Halo Assay after 24 h exposure to G3Si PEG6000 dendrimer or dendriplex. Photographs of representative cell nuclei observed during the DNA damage test, illustrating low-, intermediate-, and high-damage nuclei, with corresponding Nuclear Diffusion Factor (NDF) values of 1.57 (negative control), 4.60 (the 0.5 μM dendrimer), and 16.96 (10 μM not complexed dendrimer), respectively. The increasing ethidium bromide-stained halo area reflects greater alkali-induced DNA relaxation consistent with increased strand-break-associated damage. A blue asterisk marks significant differences relative to the control, and a violet asterisk indicates significant differences between the dendrimer and its dendriplex at the corresponding concentration (**p < 0.01; ***p < 0.001). Data are presented as mean ± SD for at least 4 repetitions. Images were acquired at 400× total magnification.

Confocal uptake imaging in the present work revealed that while most noncomplexed dendrimer signal localized outside the nucleus, a small number of entities were detectable within the nuclear compartment, indicating that direct primary interactions with genetic material are at least plausible for a minority fraction (Figure 5). Sporadic nuclear localization of cationic dendrimers has been reported previously in confocal studies, supporting plausibility of a nanoparticle induced genotoxicity.^48,49^ Given that nanomaterials can induce genotoxicity through direct and/or indirect mechanisms, the rare intranuclear dendrimer signal is best treated as a supportive context rather than a mechanistic discriminator.^50^ siRNA complexation markedly mitigated the DNA damage signal, as dendriplex-treated cells remained below 3 NDF and did not reach statistical significance, supporting the conclusion that complexation reduces the cationic carrier fraction and attenuates the pathways that culminate in strand-break readouts.

### Blood–Brain Barrier Model Integrity by Transendothelial Electrical Resistance (TEER)

To extend single-cell screening to barrier-relevant evaluations, we employed a multicellular BBB model that captures essential neurovascular unit (NVU) interactions in which barrier properties emerge.^51^ BBB properties vary across the vascular tree due to anatomical and molecular zonation, with practical implications for how nanocarrier exposure translates into changes in barrier integrity and permeability. Cerebral capillaries account for approximately 85% of the total cerebral blood vessels’ length and provide the dominant endothelial exchange surface of about 12 m^2^ in the adult human brain, indicating that most blood-to-brain interfaces encountered by circulating therapeutics are capillary interfaces.^52^ On this basis, we employed a direct-contact, triculture with capillary-weighted cellular composition, reproducing an endothelial-dominant barrier layer on the apical face of the well supported by high pericyte representation and robust astrocytic ensheathment (Figure 7). This arrangement mirrors key structural features of the *in vivo* capillary wall and preserves reciprocal BBB signaling, providing sensitive detection of compound-induced changes in barrier integrity.^53^

**Figure 7:**
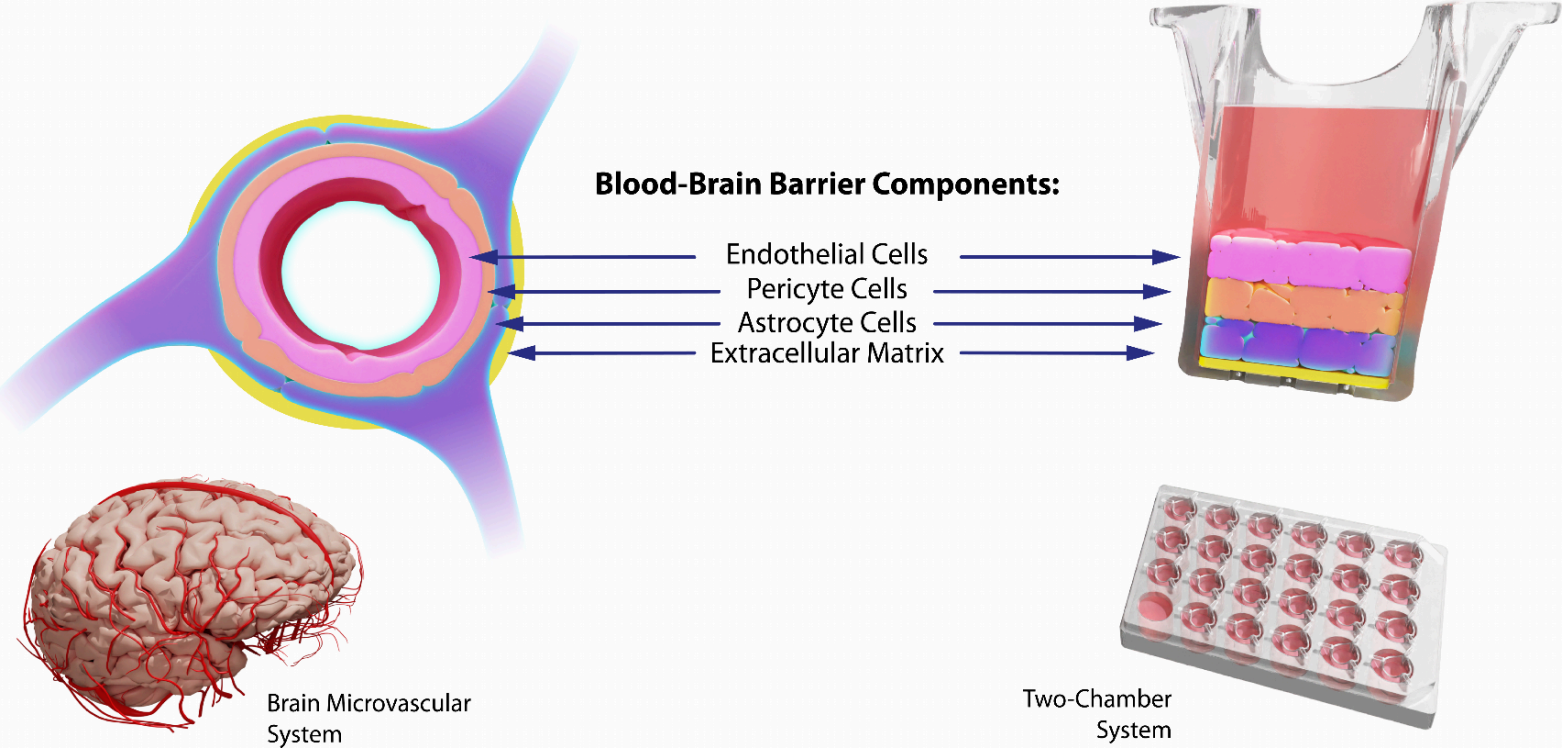
Illustration depicts a schematic cross section of the Blood–Brain Barrier (BBB) on the left and the corresponding *in vitro* barrier model established in the inset of the two-chamber system on the right.

The effects of the G3Si PEG6000 dendrimer and its dendriplex on the BBB model integrity were quantified by monitoring transendothelial electrical resistance (TEER). TEER is a widely used, label-free, noninvasive electrophysiological readout and serves as a quantitative proxy for barrier integrity *in vitro.* The approach is grounded in early *in vivo* microelectrode studies of brain surface microvessels by Crone and colleagues, and TEER has since become a routine quality-control attribute for BBB models.^54^ The measurement is obtained by applying a low alternating current (AC) conducted by small inorganic ions in the culture media between the electrodes placed in the apical and basolateral compartments. The measured resistance reflects the overall ionic resistance across the cellular barrier. Compounds were added only after the readout plateau on day 13 was reached to avoid confounding maturation with treatment effects. The not-complexed G3Si PEG6000 dendrimer elicited a concentration-dependent, nonmonotonic TEER response (Figure 8). Twenty-four h after the addition of the compounds, the highest concentration of the noncomplexed dendrimer elicited a modest, transient TEER decline (90.25% ± 6.71 of control), followed by recovery on the subsequent measurement (111.79% ± 13.02) and a progressive overshoot above baseline that peaked at day 5 (136.85% ± 26.14) and remained elevated through day 12 (124.56% ± 21.60). By contrast, at an equivalent concentration, the dendriplex did not induce an early decrease (101.45% ± 22.12 at 24 h) and produced progressive (143.32% ± 16.24 at day 5) and statistically significant sustained elevation in TEER in the later stages of the experiment (149.00% ± 9.62 at day 12) (Figure 9). Notably, none of the tested formulations produced a prolonged TEER decline.

**Figure 8:**
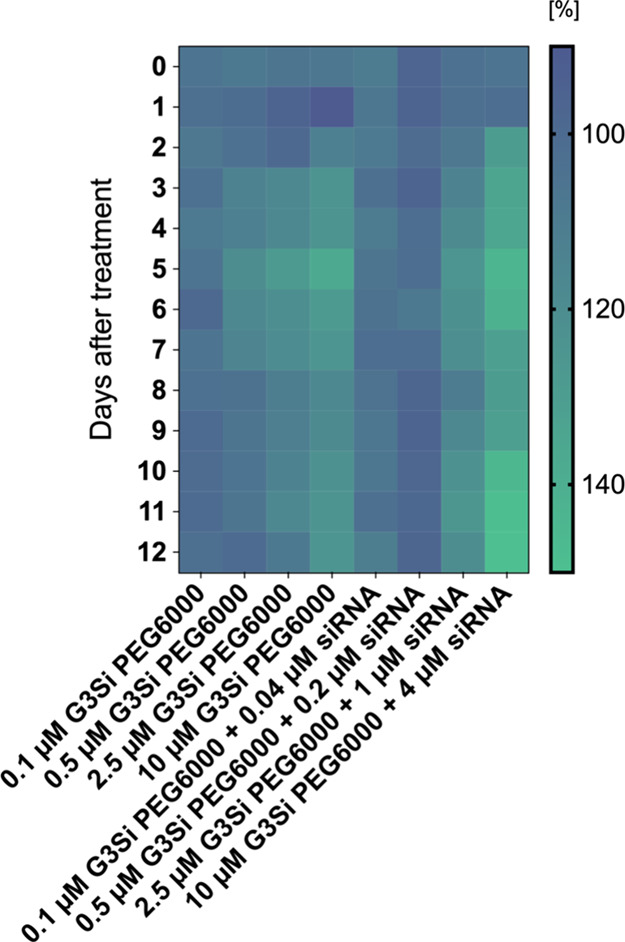
TEER dynamics of the blood–brain barrier (BBB) model following exposure to G3Si PEG6000 dendrimer and its dendriplex. Data are expressed as a percentage of the untreated control from 3 repetitions. The TEER_REPORTED_ data at days 3, 5, 10, and 12 post-treatment are summarized in Figure 9.

**Figure 9:**
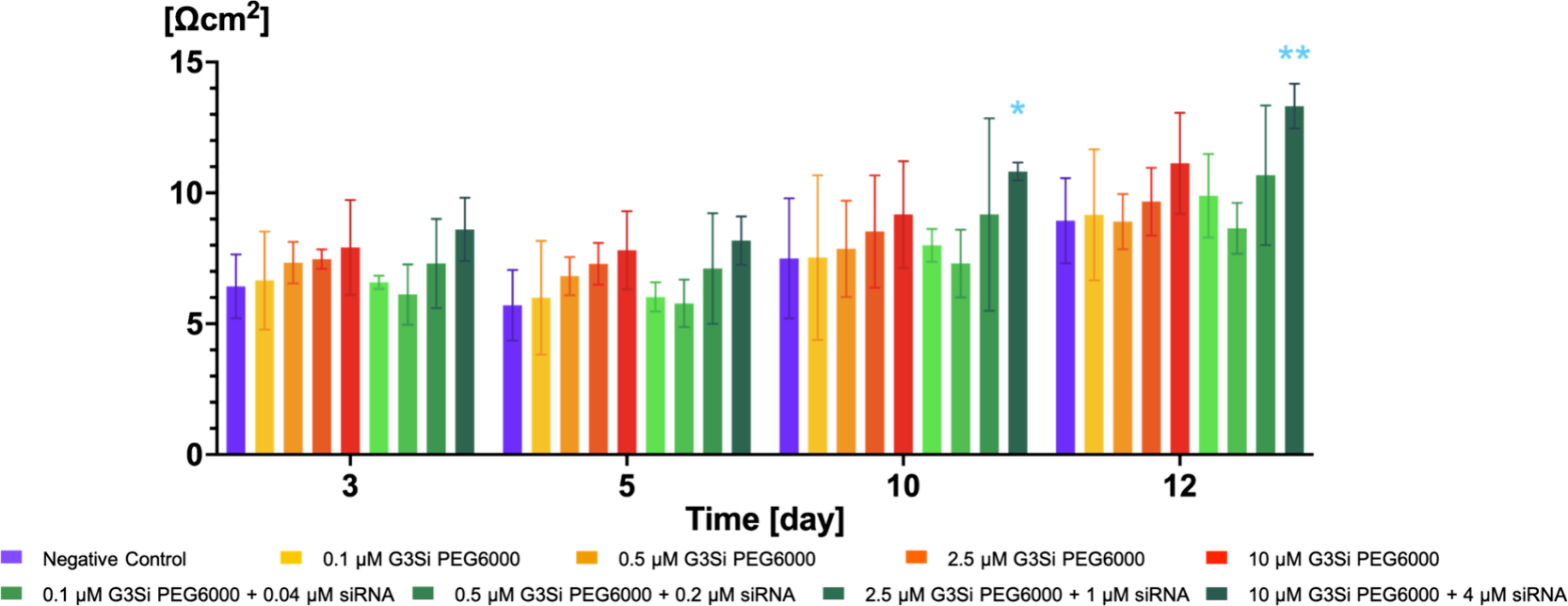
Changes of TEER_REPORTED_ [Ω·cm^2^] of the blood–brain barrier (BBB) model following exposure to G3Si PEG6000 dendrimer and its dendriplex. A blue asterisk marks significant differences relative to the control (*p < 0.05; **p < 0.01). Data are presented as the mean and standard deviation for 3 repetitions. A heatmap summarizing all post-treatment days is shown in Figure 8.

The early divergence between formulations is plausibly attributable to reduced bioavailable cationic surface activity upon siRNA complexation, which decreases the free dendrimer fraction that can drive acute membrane perturbation and stress responses. The pattern may reflects adaptive, mesoscopic remodeling of the triculture BBB, but the present data are not sufficient to determine the possible contributors including changes in intercellular adhesion, junctional organization, cell–substrate adhesion and proliferation that improve coverage of the inset surface and reduce microheterogeneity, all of which may influence the net resistive path measured with TEER.^55,56^ In the absence of direct imaging-based validation, this interpretation remains hypothetical and will be addressed in future work. Reinforcement of BBB integrity is particularly relevant in the conditions such as the AD, where barrier disruption contributes to neuroinflammation and accelerates neurodegenerative processes.^57–59^ By maintaining or enhancing barrier integrity, the dendrimer-based formulations indicate a favorable profile for preserving the barrier function.

### Blood–Brain Barrier Paracellular Permeability

The effects of the G3Si PEG6000 dendrimer and its dendriplex on BBB-model paracellular transport were quantified using a 40 kDa FITC–dextran flux assay. Fluorescent dextran tracers of defined nominal molecular weight are widely used in Transwell barrier models to quantify macromolecular paracellular permeability, and they are commonly paired with TEER because tracer flux and electrical resistance provide complementary readouts of barrier performance.^60,61^ Interpretation of these orthogonal permeability methods requires emphasizing that TEER and dextran flux, also termed the macromolecular tracer assay, provide complementary but noninterchangeable measures of barrier performance. TEER reports area-averaged translayer electrical resistance and primarily reflects net ionic conductance across the barrier. In contrast, FITC–dextran permeability reports macromolecular passage and can be disproportionately influenced by spatially restricted routes that can contribute relatively little to global ionic conductance. As the FITC-dextran data were normalized to untreated control, the results should be interpreted as relative fluorescence-based transport data rather than absolute permeability coefficients.

At day 3 after exposure, the paracellular flux data revealed an early divergence from TEER, indicating a decoupling between these orthogonal measures of barrier function. Specifically, the highest tested concentration of the not-complexed dendrimer increased macromolecular permeability to 175.51% ± 28.90 of control, while the matched dendriplex concentration increased permeability to 163.71% ± 32.40 of control (Figure 10). In the corresponding conditions, TEER was not significantly reduced, despite the increased macromolecular flux. We hypothesize that the observed decoupling reflects a nonuniform perturbation of paracellular organization, with a transient increase in a macromolecule-permissive leak pathway occurring in the absence of a commensurate change in the pathway that predominantly determines ionic resistance. This pattern is consistent with permeability being governed by spatially restricted or short-lived high-permeability routes that can disproportionately increase 40 kDa FITC–dextran flux while contributing minimally to the area-averaged TEER signal. Plausible microstructural contributors include transient bicellular discontinuities and tricellular junction-associated transport.^62,63^ Mechanistic interpretation remains tentative and should be evaluated by imaging-based quantification of junction continuity together with analysis of tight-junction marker localization and organization.

**Figure 10:**
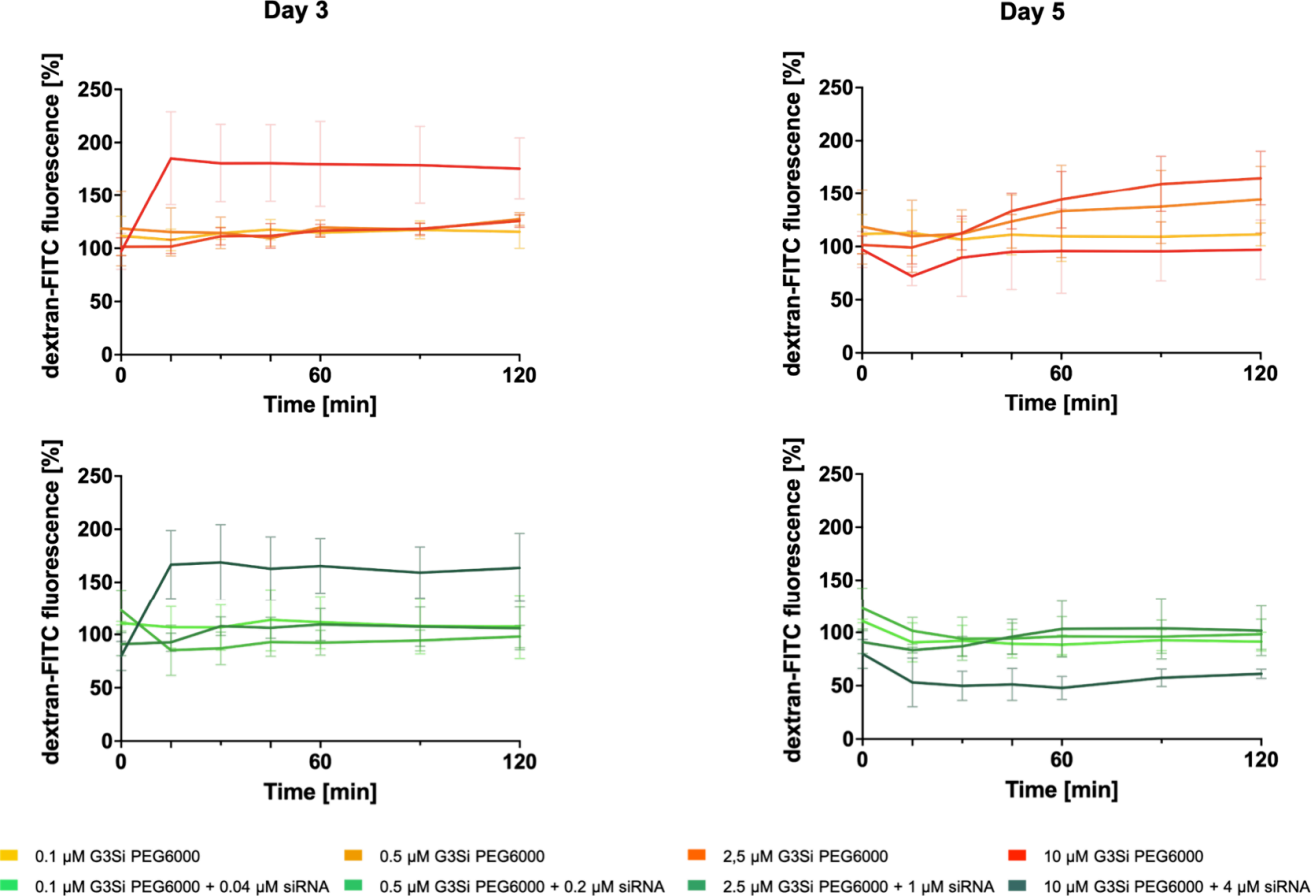
Accumulation of 40 kDa FITC-dextran in the basolateral compartment of the blood–brain barrier (BBB) Transwell model measured 3 and 5 days after exposure to G3Si PEG6000 dendrimer or its dendriplex. Fluorescence intensity in the basolateral compartment was recorded at 0, 15, 30, 45, 60, 90, and 120 min after addition of FITC-dextran. Data are expressed as a percentage of untreated control at each time point for 3 repetitions.

By day 5, dextran permeability in the highest tested concentration of the noncomplexed dendrimer returned to near-baseline levels at 97.03% ± 27.87 of control, whereas the highest concentration of dendriplex reduced dextran flux to 61.00% ± 4.37 of control, coinciding with the TEER overshoot observed at later time points. The concurrent TEER overshoot and reduction in 40 kDa FITC–dextran flux is consistent with a delayed change in barrier-associated properties after dendriplex exposure. A plausible hypothesis is that dendriplex exposure promotes the reinforcement and reorganization of intercellular junctions and endothelial adhesion, which can decrease the effective paracellular leak of macromolecules and thereby suppresses dextran passage. This interpretation aligns with findings from BBB microfluidic models, in which the tested nanoparticle led to concurrent increases in TEER and enhanced expression of junctional markers ZO-1 and VE-cadherin, signifying a reinforcement of tight junctions and a subsequent reduction in barrier permeability.^55^

At lower concentrations, short-term exposure to subcytotoxic levels of the not-complexed dendrimer preserved macromolecular permeability, whereas prolonged exposure produced a concentration-dependent increase in dextran flux. On day 5, the 2.5 μM concentration elevated dextran flux to 164.70% ± 25.60 of control, and the 0.5 μM concentration elevated dextran flux to 144.25% ± 31.51 of control. These time-dependent and concentration-dependent changes are consistent with a delayed adverse effect of the not-complexed dendrimer on paracellular macromolecular transport under prolonged exposure. In contrast, the corresponding dendriplex formulations did not increase dextran permeability and maintained macromolecular permeability at or near control levels.

Accounting for key *in vivo* determinants of BBB function that are not fully recapitulated in static transwell systems, the *in vitro* profile suggests that subtoxic dendriplex concentrations do not increase macromolecular leak or impair ion pathway function, thus suggesting they are unlikely to compromise BBB integrity *in vivo*. This prediction is particularly relevant in AD, where BBB dysfunction can be regionally heterogeneous and influenced by local vascular pathology rather than reflecting a uniform, global loss of barrier function.^64,65^

### Spatial Learning and Memory in the Alzheimer’s Disease Murine Model

Applied *in vitro* assays are indispensable for mechanism and screening, however their predictive value for CNS delivery and safety ultimately depends on how well key *in vivo* modifiers are represented. We therefore interpreted the subsequent *in vivo* study as an essential translational step positioned to test whether the formulation-dependent patterns observed *in vitro* translate into disease relevant performance in a whole organism setting.

Cognitive performance was assessed using the Morris Water Maze, a well-established test of spatial learning and reference memory in which rodents learn across repeated trials to locate a submerged escape platform in a circular pool by using stable distal cues in the testing room. Reference memory is assessed in a probe trial, during which the platform is removed, and search behavior is quantified by the animal’s preference for the former platform location. Probe-trial outcomes were summarized as time spent in the target quadrant and the number of crossings through the former platform zone, which provide complementary readouts of spatial bias and the precision of searching around the learned goal location (Figure 11).

**Figure 11:**
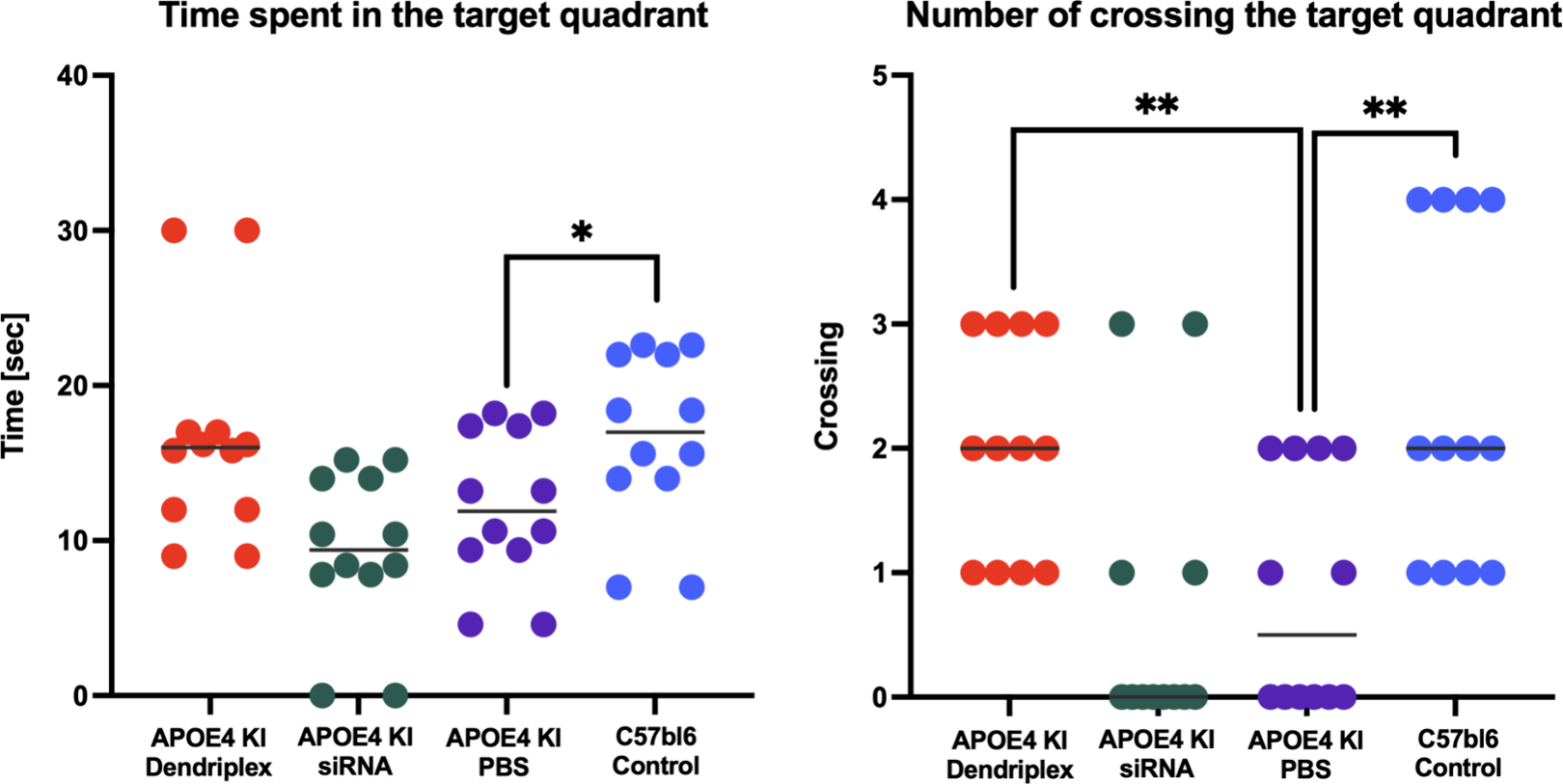
Spatial memory performance of APOE4 KI and C57bl6 in the Morris water maze probe trial. Left panel: cumulative amount of time animals swam in the target quadrant; Right panel: total amount of platform zone crossings. An asterisk marks significant differences relative to the APOE KI control (*p < 0.05; **p < 0.01). Data shown as individual values (*n* = 12) and group median (horizontal lines).

There were no significant changes in escape latencies between any of the tested groups (data not shown). In both probe-trial end points, APOE4 knock-in (KI) mice receiving PBS performed worse than C57BL/6 PBS controls, supporting the suitability of the experimental conditions for detecting APOE4-associated cognitive vulnerability relative to the healthy counterparts.^66^ In the APOE4 KI cohort, treatment with the G3Si PEG6000 dendriplex resulted in a significant increase in platform-zone crossings compared with the APOE4 PBS group. In contrast, only a trend toward increased time spent in the target quadrant was observed in the APOE4 KI group, and the accompanying rank-based effect size (r_rb_ ≈ 0.22) indicated only a modest shift with substantial overlap between group distributions, consistent with a partial or heterogeneous behavioral benefit rather than a robust normalization of sustained spatial bias. This dissociation is plausible because crossings reflect how precisely the animal searches the small spatial region, whereas quadrant time is a broader measure that can be diluted by mixed search strategies variability between animals, including noncognitive contributors that are well recognized in water maze probe analyses.^67,68^ This pattern is consistent with published reports on APOE4 knock-in mice, in which Morris water maze deficits are generally modest and end point-dependent rather than uniformly detected across all measures.^66^ This pattern is consistent with the broader literature indicating that APOE4-associated cognitive dysfunction emerges early but remains relatively subtle and end point-dependent in young APOE4 targeted-replacement mice, whereas more robust and reproducible probe-trial deficits become apparent in middle-aged animals.^69,70^

Collectively, the dendriplex profile is most consistent with a partial improvement in spatial search precision in APOE4 KI mice, which supports the premise that formulation-mediated delivery can translate into measurable functional benefit. Yet, the behavioral assessment relied on a single task, these findings should be interpreted as preliminary functional evidence rather than definitive proof of broad cognitive improvement. By contrast, APOE4 KI mice receiving naked siRNA showed no improvement across probe end points, consistent with well-established *in vivo* limitations of unformulated siRNA, including rapid nuclease degradation and rapid systemic clearance, which together reduce tissue exposure and cellular internalization. A nontargeting (scrambled) siRNA control was not included in the present *in vivo* design. Accordingly, these behavioral findings cannot be attributed specifically to APOE4 silencing and should be interpreted as exploratory treatment-associated effects under the present formulation conditions.

## Conclusions

Clinical progress in Alzheimer’s disease has advanced without a widely transferable approach that reliably delivers predictable, compartment-appropriate CNS exposure under BBB constraints, reflecting both momentum and persistent limitations in neurological disease interventions. The sustained entry of biologics into CNS trials suggests that clinical feasibility is often achieved by accepting constrained exposure or relying on routes that are practical only in specific contexts, rather than indicating that the broader delivery challenge has been resolved. As mechanistic insight increasingly incorporates neurovascular biology and disease-associated BBB remodeling, greater emphasis is being placed on BBB architecture, transport-route constraints, and safety–performance trade-offs at the BBB interface as design inputs, alongside evaluating performance with functionally meaningful end points. In this study, we used a stepwise progression of models to link cellular responses, barrier integrity metrics, and *in vivo* outcomes to assess an RNAi nanosystem within this broader translational framework.

At the cellular level, the dendrimer served as an efficient delivery vehicle, significantly enhancing intracellular siRNA accumulation compared to free oligonucleotides. While the noncomplexed carrier signal was mainly extranuclear with rare puncta overlapping the nuclear compartment, the dendriplex showed both carrier and siRNA fluorescence, only restricted to the cytoplasm, consistent with delivery into the mechanistically relevant domain for RNAi. DNA damage screening by Fast Halo Assay showed a pronounced, dose-dependent increase in NDF following exposure to the noncomplexed dendrimer. Because FHA can also capture DNA fragmentation associated with apoptosis or cytotoxicity-related DNA relaxation, interpretation at concentrations near or above cytotoxicity thresholds requires caution; however, the presence of a clear FHA signal already at 0.5 μM noncomplexed dendrimer suggests that the response cannot be explained solely by widespread overt cell death. Importantly, siRNA complexation attenuated the FHA signal, with dendriplex-treated cells remaining below ∼3 NDF and not reaching statistical significance. This pattern is consistent with the fact that complexation reduces the bioavailable cationic fraction and thereby limits damage pathways that culminate in strand-break readouts. Rare intranuclear carrier signal observed by confocal microscopy provides plausibility for direct nuclear interaction in a minority fraction.

In a capillary-weighted, direct-contact BBB triculture model, neither formulation caused a sustained decline in TEER. The noncomplexed dendrimer produced a nonmonotonic TEER trajectory at the highest dose, with an early transient decrease at 24 h, followed by recovery and overshoot. In contrast, the dendriplex avoided the early TEER drop and exhibited a progressive, statistically significant, sustained increase in TEER at later time points. Macromolecular flux assays refine interpretation by demonstrating an early decoupling at day 3, where both the highest tested noncomplexed dendrimer concentration and the matched dendriplex increased dextran permeability without a significant TEER reduction, indicating a functional decoupling between ionic resistance and macromolecular leak at this early time point. By day 5, the highest tested dendriplex concentration reduced dextran passage to 61.00% ± 4.37 at 10 μM, whereas lower dendriplex concentrations remained at or near control levels. Dextran permeability in the highest tested concentration of the noncomplexed dendrimer returned to near-baseline levels, whereas subcytotoxic noncomplexed dendrimer increased dextran flux (e.g., 164.70% ± 25.60 at 2.5 μM). Collectively, these results are most consistent with a broader functional window for the dendriplex in barrier-relevant conditions and supporting a later shift toward reduced macromolecular permeability, while also indicating that early, spatially restricted macromolecular perturbations remain plausible and warrant direct junctional imaging and marker quantification.

*In vivo*, Morris Water Maze probe-trial analysis demonstrated that the APOE4 KI mice cohort treated with dendriplex exhibited a significant increase in platform-zone crossings, whereas time spent in the target quadrant increased only numerically and did not reach statistical significance. This dissociation is most likely due to platform-zone crossings being more sensitive to precise search focus, while quadrant time can be diluted by heterogeneous strategies and noncognitive contributors. Definitive mechanistic linkage will require brain exposure measurements and target engagement assays. Nevertheless, the functional signal observed with dendriplex treatment supports the premise that formulation-dependent delivery properties can translate into measurable behavioral benefit in a disease-relevant setting, yet the observed effect cannot be attributed specifically to APOE4 silencing and should be interpreted as exploratory. Direct brain biodistribution and knockdown evidence are required to connect phenotype to mechanism.

Concluding, the results support the hypothesis and position the position the G3Si PEG6000 dendriplex formulation as allowing intracellular siRNA uptake with cytoplasmic distribution, attenuated genotoxicity signals relative to the free carrier, and BBB-model compatibility characterized by favorable change in barrier-associated properties with a time-dependent tightening signature.

## Abbreviations

AC: alternating current
AD: Alzheimer’s disease
AGS: astrocyte growth supplement
AP: amyloid precursor protein
APOE4: apolipoprotein E4
Aβ: amyloid β
BACE1: beta-site APP cleaving enzyme 1
BBB: blood–brain barrier
CNS: central nervous system
ECGS: endothelial cell growth supplement
FBS: fetal bovine serum
FHA: Fast Halo Assay
GalNAc: triantennary *N*-acetylgalactosamine
IATA: integrated approaches to testing and assessment
ITS: integrated testing strategies
KI: knock-in
MFI: mean fluorescence intensity
MWM: Morris water maze
NDF: nuclear diffusion factor
NFTs: neurofibrillary tangles
NMDA: *N*-methyl-d-aspartate
NVU: neurovascular unit
P/S: penicillin–streptomycin
PBS: phosphate buffered saline
PEG: polyethylene glycol
PGS: pericyte growth supplement
PLL: poly-l-lysine
RNAi: RNA interference
TEER: transendothelial electrical resistance
